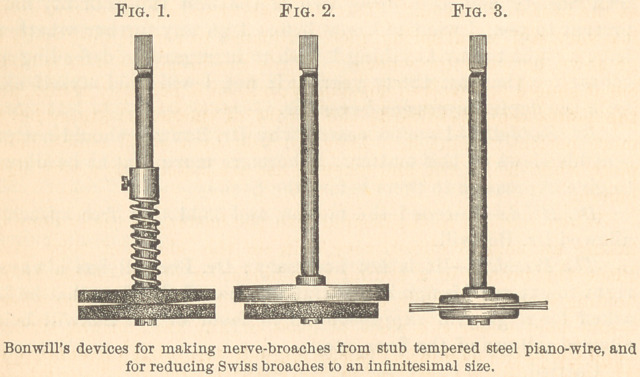# Odontological Society of Pennsylvania

**Published:** 1889-08

**Authors:** 


### ODONTOLOGICAL SOCIETY OF PENNSYLVANIA.

THE regular meeting of the Odontological Society of Pennsylvania was held Saturday evening, June 1, 1889, at the hall, Arch and Thirteenth Streets. President James Truman in the chair.

The subject announced for the evening was Patents, by W. G. A. Bonwill, D.D.S.

*Dr. Bonwill.*—It has been my intention for the past four years to bring before you what I have to present to you to-night; but before making this presentation I wish to have a fair understanding with this Society. I have brought one thing here that I do not propose to give you outright, but will ask you what I shall do with it,—whether I shall give it to you, or patent it, and make money out of it. I have tried the dental profession in many ways, and I suppose they have been very much tried with me, but I think it is not too late to have a fair understanding; and in this agreement with you I want the liberty to take care of myself with regard to my inventions, on which subject there has been so much said lately *pro* and *con.* I wish to explain to you how I have always stood regarding patents; and then, if my conduct has not been consistent with the ethics of this Society and the general ethics of the dental profession, I shall myself present a charge against myself, so that it may be decided, gentlemen, whether I have acted right or wrong in this matter. My object is for you to answer me if I have erred. On the other hand, if there is nothing I have done, then please be men enough to say so and publish it. I have certain rights in my own Society, and as I bring two of the best things of my life to present to you, I want to know before I go any further whether I have a right to say anything I wish to in regard to defending my course for the past thirty years. If not, I will hold myself aloof from the dental profession hereafter.

*Dr. Sudduth.*—I see no reason why Dr. Bonwill should not present his views on this matter. I therefore move that he be allowed to give expression to them before the Society.

*Dr. Head.*—I second the motion, and hold that free speech be allowed Dr. Bonwill.

*The President.*—It is not necessary; Dr. Bonwill has always a right to express his opinions. But in view of the fact that he has asked for a general expression, all in favor of Dr. Bonwill being allowed full use of the floor, etc.

Carried.

*Dr. Bonwill.*—I would ask, further, that these proceedings be published either in the INTERNATIONAL DENTAL JOURNAL or *Cosmos.*

*Dr. Sudduth.*—They will be published in the INTERNATIONAL.

*Dr. Bonwill.*—I have brought to you to-night three things: 1. A little instrument,—one of the most important I have in my office,—a nerve-broach reducer. Very frequently in getting into the finest pulp canals you cannot secure a suitable broach in the market for the purpose; nor can you make one by the most careful filing with anything like satisfaction. Three or four years ago, by a mere accident, I placed two corundum wheels on my laboratory lathe,—simply one small one that had been worn on one side at a slight bevel, and a little out of line, merely as a nut to hold the other,—leaving a slight space between them, as in cut. It immediately struck me that by placing a coarse corundum wheel and a soft rubber disk on the same mandrel, and screwed together, you could rasp down a broach to any desired size. By experimentation I found it was better to use two shellaced paper disks, and to have on either side of these a disk of soft rubber, so that it will keep these disks so close together that in passing your fine wire in between them while they are revolving rapidly, you can cut the broach down to an almost infinitesimal size,—the finest you can cut steel. I have never had anything that has given so great satisfaction; Swiss and English makes both failed me.

For reducing steel piano-wire, evenly tempered, use two corundum wheels kept together by a coiled spring (see cut), and afterwards use paper disks with the soft rubber on either side, for fine work.

FIG. 1. FIG. 2. FIG. 3.

Bonwill's devices for making nerve-broaches from stub tempered steel piano-wire, and for reducing Swiss broaches to an infinitesimal size.

DESCRIPTION OF APPARATUS.

No. 1 consists of two sharp-cutting corundum wheels, used dry, with one at a slight bevel, as shown in cut. The outer one is screwed fast to mandrel made for the purpose, and the inner one is shellaced to a thin sleeve, which moves lengthwise only on the mandrel, and is controlled by a coiled spring, the tension of which is regulated by the movable collar and screw on the mandrel nearest the chuck of engine hand-piece or lathe. The collar holding the corundum wheel is prevented from turning on the shaft by a pin running through it, and on a flattened surface of mandrel. This is for heavy work of reducing tempered stub wire, but will do so to a very fine broach.

No. 2 is a shellac and corundum wheel and a soft rubber disk of plain packing rubber on the inside next to the hub on mandrel, and both secured tightly to keep from turning. The rubber disk should be of greater diameter to allow the broach to be guided in between them easily. The edges at the periphery will be slightly apart when screwed up tightly. Both revolve at the same time.

No. 3 is made of two one-inch shellaced corundum paper or cloth disks, coarse or fine,—better medium for the last, which is used for very delicate work,—and on both sides of these a half- or three-quarter-inch soft rubber disk, cut from packing rubber and placed around; all screwed tightly on mandrel. There is no trouble in making them from the material every dentist has on hand, if it be only the paper disk.

In making No. 1 the bevel should be very slight and perfect, as the broach is so small. The inner wheel should have controlling it a very stiff spiral spring, nicely adjusted, to permit the broach to go down between the wheels. While the broach or wire is being cut it should be revolved constantly to keep it perfectly round, and the point kept in towards the centre of the wheels, in order to more perfectly point it. They are held by a small chuck hand-piece while being cut, and when in use are fastened to a very light handle.

When reduced to the desired size the barbing is easily done by taking an enamel chisel, very thin on the edge and very hard. Place the broach in a sliding tube-holder or chuck—same as for holding it securely while cutting down with the corundum wheels—and hold under it a piece of glass or any hard, bright surface. Hold the chisel at ninety degrees angle to the broach, commencing near the point, and with the blade of the chisel held on the broach, not directly at right angles with it, but with the right edge or corner of chisel turned farther away from it, so as to throw the barb up the right side of its length, in order to insure its catching the pulp. If cut directly across, at right angles, it will not do so nicely. A slight pressure on the chisel will raise a barb—be it ever so slight—that, if cut to the right, will be sure to catch the pulp every time. Do not make more than three or four barbs, and close together at the point, and not so deep as to endanger its main strength. This can be very successfully done by a little practice, and should always be made at your leisure, that it be not hurried and best results follow, which can only come from a delicate and experienced touch.

2. I made about two years ago, when Dr. Woodward presented his new matrix, the appliance I present to you here to-night. It is applicable in a great many cases. It is easily adjusted, and you can make a very perfect operation on marginal cases, and it can also be removed without difficulty. It is adapted for gold or amalgam, and allows of the matrix being removed without interfering with the perfect contour of the filling, which can be made in this way only, since it fits snugly the cervix, and very little, if any, trimming will be needed there afterwards. When there is a cavity opposite to the one being filled it should be filled with pink gutta-percha to back up the divided matrix and prevent it springing back into the cavity. Each jaw of the matrix is double, and can be turned end for end. It is also shaped on both sides, that cavities in adjoining teeth can be filled without change.

This matrix will be more easily understood when I tell you to shape, either in brass or hard rubber, a piece to fit perfectly the buccal side of a bicuspid or molar, and made to run half-way through; and another from the palatal or lingual side adapted thereto, and also running through to meet the one from the buccal side. These, shaped nicely to the contour of each tooth, are now attached to a clamp such as is used for rubber dam, to a universal clamp by a thread and screw, to allow the jaws to adjust themselves to the surfaces. These jaws can be unscrewed and others substituted.

3. The third invention is something to which I wish to ask your serious consideration,—whether I shall patent it or present it to the dental profession. It is a right-angle plugger, and as important in its way for either the electrical or mechanical mallet as the right angle is to the hand-piece of the dental engine. Last January, when surrounded by my family at dinner, the idea of this instrument flashed in my mind, and I knew at once, without trial, it would prove satisfactory. I had long felt the need of a right angle for my pluggers, and this accomplishes it as perfectly as if it were simply filling in a straight line. With it I see no further change needed in these instruments. It fills a gap not hitherto approached. Many cavities heretofore that should have been filled with gold were made with amalgam. It is this I now offer you for a verdict. But before presenting it I desire to speak of my position as a patentee. You must all grant there are two sides to every question, and this one is no exception to the rule. A great effort was made by Dr. Merriam, some time ago, to have it understood that we cannot be professional unless we give unreservedly everything to the dental profession, no matter what we get up and what it costs us in the shape of time, money, etc. His arguments were merely assertions, and they have no weight unless backed by evidence. Personally, I have but two things for which I feel answerable, and these I will mention. Before I had much experience, and while in Delaware, still circumscribed by unfavorable surroundings, with very little encouragement, no dental society, and in communication with only one dentist in the State, on the spur of the moment, in my experiments, which I commenced early in my career, I thought of electricity for the purpose of removing nerves painlessly and obtunding sensitive dentine. I had no sooner patented this than I saw I had made a mistake. I never mentioned it as a patent afterwards. I found it good, but experiment gave me something better,—rapid breathing as an obtunder. I gave all to dentistry. The only other incident in my career for which you can censure me, as I look at it, would be when I came here to live and placed my electric mallet and dental engine on the market. (I have never asked a man to purchase one of my inventions.) After that I had received many strong testimonials from Drs. Darby, Guilford, and others, which I published in a circular, placing it on the stand with my exhibit in the Franklin Institute, where I was afterwards awarded a silver medal. In it I merely stated the virtues of those instruments. I was afterwards sorry for doing it, as some of the gentlemen took exception thereto. It was not done with any bad spirit, or to make practice, for at that time I had an income that would surely keep the wolf away. I only thought it was right to give to the world the testimonials in favor of the instrument used by leading dentists.

I have not patented everything that I have made. Numerous things have I gotten up which remain unpatented; one of the most important of which, when it is understood, is the "anatomical articulator." It was the first thing I ever did, and I gave it to the dental profession at the meeting of the American Dental Association, through Dr. J. H. McQuillan, and I do not think the proceedings show that I was even thanked for it. It was not discussed; it dropped flat. Another instance I will mention. I went to Saratoga to the American Dental Convention, and presented there the appliance that so much has been made of since by Dr. Talbot, of Chicago,—the "spiral spring" for correcting irregularities. A man sitting in his shirt-sleeves (and no gentleman would do this in the presence of others) abused me most personally for presenting such a thing as that, even as a gift. I felt like going through a knot-hole. If anything would have dampened my energy, and put a bad spirit in me to never present myself before a dental society again, it would have been the action of this ignorant person. However, it did not stop me, as results since show.

Now, if you will look back you will see a number of appliances belonging to my engine that were brought out through my instrumentality by Dr. Arthur, of Baltimore, and at my suggestion, as his letter to me shows, the shellaced disk was made; yet I made no effort to patent it. The different corundum points, known as the Northrup forms, were gotten up at the same time as my dental engine,—in 1870. (The first engine put on the market was in 1871.) Then came the soft rubber disk, the hard rubber corundum disk, and the soft rubber corundum disk; rapid breathing as a pain obtunder in the treatment of sensitive dentine and extraction of teeth, which Brown-Séquard, of Paris, and other physiologists say was a new discovery and practicable; the diamond drill in dentistry and surgery, for dental engine; the pointed fissure burr to do away with the file in separating teeth for filling and anticipating decay, and finishing gold and amalgam filling at the cervix.

The first right-angle attachment to the dental engine was made and given in 1873, which was well worth a patent; the use of Japanese bibulous paper for pressing surplus mercury from amalgam fillings when placed in the cavity, which was as much of an advance in that line as was cohesive gold.

The little apparatus I now give you for reducing nerve-broaches from wire to an infinitesimal size cannot but be of universal use, as nothing before filled the breach.

Lastly, which should have been among the first, the application of a specific oval face to the pluggers for the electric and mechanical mallets, like the face of a gold-beater's hammer, which spreads laterally while packing in a straight line. This alone was enough. When realized and practised it will forever revolutionize the packing of gold by "wiping it in." It reduces to a certainty that gold can be almost welded to the surface of a tooth. It saves two-thirds the time and nearly all the labor, is easier to the patient, and prolongs the operator's life. When Herbst saw it he at once acknowledged that his method was anticipated years before.

One more thing given: Abbey's old-fashioned soft gold foil was, considered of no use under the mallet. For the first time I reversed the decision. In 1870 I demonstrated with the electric mallet it could be used with smooth oval-faced pluggers, and welded the same as cohesive gold. It is no longer doubted. It made soft foil equally valuable and more so than cohesive gold, and the discovery was paramount in importance to all my others.

For the first time in the history of surgery I applied the diamond drill to remove a stone two inches in diameter from the bladder of a female, using my surgical engine, with the many appliances connected with it.

The jack-screw for elevating fractures of the skull instead of trephining was an invaluable affair, and patentable.

All these I have made no effort to secure by patent. They are gifts. Are these not enough?

I have always considered that anything a dentist can make should be freely given; but when it comes, gentlemen, to instruments that are complicated and applicable to mechanical pursuits, then, when you attempt to step upon a man who patents a thing that is not only adapted to the dental profession, but hundreds of other purposes, you make a mistake. When it comes to instances where a man cannot make a thing himself, he will have to go to the manufacturer anyway. Take alone the electric mallet. Use can be made of it as a hammer for cutting stone, and for a great many other purposes. Was it right that I should simply give it away? Should I not go to work to improve and patent it, and then, if it was particularly applicable to dentists' use, could I not exercise the right the government gives me to put my money into it? Nearly two years expired before I patented either the dental engine or the electric mallet.

You all want to get everything as cheaply as you can. Now, when it comes to machines, to get these up to sell at anything like a low price, it must be done by the hundred or thousand. The more you order the lower the price will be. Surely somebody ought to be protected in their manufacture if you are to get these articles at a price fitting your pocket-book. I felt that, inasmuch as I had not the money to make these instruments as they ought to be, it would be perfectly right to put them in the hands of the Dental Company, which would put its money in it, which was done after making them practically a perfect success.

A gentleman who was just from the dental meeting in New York had the good feeling to inform me that I had degraded the dental profession more than any other man in it, simply from the patenting of my inventions and selling them out to a manufacturer. I thanked him. He said my inventions were the best on earth, and I had sold them to him at a reduced price. Now, in the manufacture of these articles, if you made them yourselves you would soon find out the difference. They will not only be imperfect, but they never would be perfected. So, in my experience, if I had the ground to go over again, I would do the same thing.

The most potent argument I can give you as to the impracticability of giving away inventions when first made practical is in the history alone of my own inventions.

The anatomical articulator was given to the profession, and it took dentists twenty-eight years to grasp the facts demonstrated.

In eighteen years there has not been a valuable improvement placed on my dental engine, electrical or mechanical mallets. The high price paid me for them tells their value. What would have resulted had I given them ? If dentists, with capitalists and manufacturers on their side, have not proven equal to the emergency, how long would these instruments have been kept in obscurity?

When we have in our ranks better mechanicians and more inventors it may be well to give away ideas and let others do the work of invention and improvement, and let many share the honors, which would be found "very easy." Any one entering into the manufacture of a new thing must go against *the tide.* Long years of fighting in introducing any new article must have backing. The experience of men in the past has led to laws which will secure not only an individual, but a nation, in the outlay made; and will do so for a number of years, until it will stand on its own feet and has arrived at manhood. If men and corporations could not have such protection, there would be slow progress, if really it would occur at all. Let any of you turn inventor, or even improver, and at once you will see whose ox is gored. You who know nothing of the practical workings of such matters cannot conceive what is involved in such work. Come to my museum of inventions of thirty years' duration, and you will tell me I have done right to secure myself against dentists and manufacturing companies, and made what I have in money and honors. If battling for a principle is correct in any way it is doubly so here, from the long line of work, and in many fields, that it has been my fortune to have placed before me. The law of preservation demands that we shall look after and rear our own children. There is injustice in the workings of all laws. We find it so in patents. It will always be so. Let us divide it and give to the originator what is his due, and which he will have if he has the right spirit. Such communistic ideas are not in keeping with the democratic age, and with all your "Dental

Protective Associations" you cannot down the spirit of an inventor. The wheels of time would stop and turn backward. Listen to me and for once stop to consider, and you will learn to be just.

These are the principal points I wish to make in regard to this in a business way. Another thing. Instead of making so much fuss about the Low crown and fighting it, as I told them the other night, they would do themselves infinitely more credit if some of them would look into the matter and bring out a better result. You would find very readily that you would not infringe upon it. Do you not know that there has hardly ever been anything devised but that somebody has improved upon it to a certain extent, and many supposed generic inventions blotted out?

DISCUSSION.

*Dr. L. A. Faught.*—I am very much interested in the instrument Dr. Bonwill has exhibited to-night. It seems to have only one fault,—the noise of the engine in packing the gold. There seems to be the same objection to it, in this respect, as to the right-angle hand-piece. Of course, the noise of an instrument of that kind on a very nervous patient would not be pleasant. It has excellent power, and undoubtedly does the work.

*Dr. Bonwill.*—The blow can be regulated. Much has been said about the noise of the mechanical mallet. Dr. Register thought that a rolling lug would do away with it. It is not, however, the contact of the projection on the wheel that makes the noise. A patient will tell you the noise is in the mouth itself where the plugger comes in contact with the gold.

*Dr. D. N. McQuillan.*—I want to say that to-day I put two people to sleep with the mechanical mallet.

*Dr. Joseph Head.*—I have frequently done the same thing.

*Dr. W. X. Sudduth.*—As I understand Dr. Bonwill, he will not give this plugger to the Society except the Society agrees to manufacture it and put it on the market.

*Dr. Bonwill.*—That is correct.

*Dr. Sudduth.*—In regard to patents, I think that I have made my position very generally understood on the subject heretofore, but will again say that I hold that the patenting of an appliance that has a use outside of dentistry, such as the principle involved in the electric mallet and such like instruments, is perfectly justifiable; also, that in case a dental instrument is complicated and requires the expenditure of capital to perfect and manufacture it, that in such cases the inventor has a perfect right to secure himself and those connected with him, or those to whom he sells his invention, from unjust usurpation of his or their rights as original discoverers and perfecters of said invention; and that a dentist so doing should not be considered unprofessional for his conduct. In the case of such an invention the manufacturer places a finished product upon the market,—an article of commerce that is ready for use, such as a dental chair, engine, mallet, etc. But in the case of the Low crown and bridge-work patents an entirely different condition arises. A principle or method is patented, and the profession is compelled to assist in its application, and a royalty is demanded for the use of what should be free as air. Such patents are an outrage upon the profession, and he who will stoop to enforce them should be ostracized by all regular societies throughout the country. In the same category should be placed all patents on simple contrivances such as can be made by the dentist in his laboratory, and all secret nostrums. This should be done not alone for the benefit of the profession, but for the good of the individual himself.

*Dr. Bonwill.*—What are your personal views in regard to Dr. Merriam's position,—that all men who do patent are to be ostracized from societies on the ground of unprofessional conduct?

*Dr. Sudduth.*—I have said that if dentists want to be recognized by the medical fraternity they must comply with the code of ethics of the American Medical Association. The professional phase of the question has already been discussed.

*Dr. Bonwill.*—Let me explain, and show how foolishly you are acting. Only a short time ago, at the last meeting of the medical men at Washington, Professor Harshley, of London, who has performed a great many operations, instead of feeling injured, or that the profession of medicine was lowered in its tone, had the kindness to say before the Society regarding the surgical engine which I left when I was on the other side, "This to one of your fellow-countrymen. Were it not for the surgical engine the operations that I have performed to-day could not have been very well performed." The best words I have received are from the medical profession. Professor Gross did not consider it lowering at all to have me stand by and perform operations he could not do. Not only that, but he has placed a cut of my engine in his medical work, where he speaks of it in the highest terms. If medical men notice in that way patented articles, and also men of such note, why should men who are attempting to attach themselves to the medical profession have anything to say against it?

*Dr. Tees.*—If you will look over the old dental journals and examine the matter, I think you will find that many things that have been given to the profession and published are now hidden away in the dark. Had they been patented and manufactured by the inventors, dentists would have been benefited by them. But little is now heard of the lancet beak or subalveolar forceps which I invented and presented to the profession in 1875; while the liliput furnace, that I patented and made it a special duty to introduce, is now in use by dentists all over the world. Who are the most useful among us, men like Dr. Bonwill, or those who are trying to exclude such as he from our dental meetings? They have been trying to do it for the past thirty years; they have tried to have oppressive ethical laws adopted by the Pennsylvania State Dental Society and by the American Dental Association, but the majority are opposed to them; they are not tolerated by these societies. At a late meeting of the New York Odontological Society, which has been hitherto conservative, the subject was again agitated in an essay and discussion. In the June number of the *Dental Cosmos*, in which the proceedings are published, Dr. White presents an able and sensible editorial upon them.

*Dr. Joseph Head.*—I think in a case like this, when the subject of patents is being discussed, every dentist should frankly state his opinions; for when any class of persons is reproved by popular opinion, it is only just that the adherents of that class should all stand together and express their sentiments.

A man who invents methods, the patenting of which cripples dentists in following out their own individual operations at the chair, does a great injury to the profession at large; but the man who expends his time and energies in developing the profession by inventing articles which must be sold, and which can with profit be used by dentists, I think has not only a perfect right to be remunerated for his time, but also a right to honor for his industry.

*Dr. James Truman.*—As has already been stated, it is important that each one should give as clearly as possible the position he occupies on this question. As Dr. Tees has stated, the subject has been a cause of contention for many years; but, for reasons easily understood, has recently assumed an aggressive character out of proportion to the matter involved. It is not a question of ethics, but is simply one of property, and it should, it seems to me, be discussed in that light and no other.

When an idea is conceived by an individual it requires time for its development. Months, perhaps years, must be consumed, together with a large amount of money. Now, this is capital invested as truly as in any other business. After thus investing his thought, skill, and means, is the profession to step in and say to this man, "You must deliver that to us. The code prohibits you from receiving anything but honor?" Such is practically the substance of all that has recently been said upon the subject. Is not the statement too absurd to be the principle of action of a generous profession?

If Dr. Bonwill were to give the right-angle plugger he has exhibited here to-night to the profession, the result would be that every manufacturer would have it without cost; but do you suppose the dentists of the country would get it one dollar cheaper than right-angle drills are at present sold? The matter would be entirely in the hands of the dealers, and neither Dr. Bonwill nor the profession would reap the slightest benefit.

The medical profession is constantly brought up as an example. I know of no reason why we should follow that conservative body; but in their case they make use freely of patented instruments, and I doubt if they ever give it a thought.

I, perhaps, go further than some on this question, inasmuch as I believe it not only the right but the duty of every man to patent anything coming within the scope of the law. The profession, as such, has no more claim upon the product of a man's time and skill than a person has to demand of him professional services without remuneration.

Again, I hold that for the security of the "*honor*" so much talked about, a patent is the one essential thing. Without it he will most assuredly be robbed of the credit of the invention, as Dr. Tees was of his, and hundreds of others besides. We must accept the morality of the world as we find it, and not as we would prefer to have it.

This is not only true of appliances, but it is true of ideas. Some men say, as Dr. Merriam has contended, that when a man has an idea worked out he should give it to the profession. I would make this voluntary on the part of the individual, but I would call to a severe account the man who deliberately embodies an original idea in book form without due credit. If it were possible to get original ideas registered, I think it would be an excellent thing. The extent to which this sort of robbery has gone is not appreciated by the profession. A man gives the thought and work of months, embodying original investigations; the idea of money, or even honor, is not the incentive to action. He publishes the result in one of the journals. The next book that appears will in all probability contain his ideas, if not exact words, in its pages without quotation-marks, and on the title-page the author (?) will refer to this gentleman as one from whom he has freely quoted. The next author will quote the preceding writer as authority for his statement; and in the course of years it seems a puzzle for honest writers to find the original man who formulated the thought into actual practice. This has been done over and over again, and there has been no prominent original writer who has not suffered in this respect.

I hold, as I should have said before, that every man must do the work as it seems best to him. If he feel it right to patent an invention, he should be allowed the liberty to do so, and not feel it necessary, as Dr. Bonwill has this evening, to come before us for our endorsement. I do not think there is a member of our profession anywhere but could say of him that his work in our profession has been equal, at least, to any other man in it, and that he should receive every honor for it.

*Dr. Sudduth.*—As I understand it, the right-angle mallet is to be used in conjunction with the Bonwill hand-piece?

*Dr. Bonwill.*—That is correct.

*Dr. Sudduth.*—I think some action ought to be taken regarding this right-angle attachment. It is not right that it be passed by without some action.

*Dr. D. N. McQuillan.*—It seems to me that we have a chance now to put ourselves on record as a Society. Dr. Bonwill has very generously offered us this instrument, and if by any means we can put it before the profession, we ought to do so. Here is a chance to do what every one has been looking for.

*Dr. Tees.*—I move that it is the sense of this meeting that Dr. Bonwill shall use his own judgment in disposing of his invention. Societies often have considerable influence in these matters. The treatment of Low by the Illinois State Dental Society forced him to sell the bridge patent. His paper describing his invention was refused publication by the Society on the ground that the writer was not "ethical." Instead of this kind of treatment we ought to encourage men with the right spirit.

Carried.

*Dr. Sudduth.*—I move that the thanks of the Society be tendered Dr. Bonwill for the instruments he has so freely given the Society this evening.

Motion unanimously adopted.

## Figures and Tables

**Fig. 1. Fig. 2. Fig. 3. f1:**